# A structural equation modelling of the buffering effect of social support on the report of common mental disorders in Zimbabwean women in the postnatal period

**DOI:** 10.1186/s13104-019-4151-1

**Published:** 2019-02-28

**Authors:** Tanaka Kaseke, James January, Catherine Tadyanemhandu, Matthew Chiwaridzo, Jermaine M. Dambi

**Affiliations:** 1 Department of Rehabilitation, University of Zimbabwe, College of Health Sciences, P.O Box A178, Avondale, Harare, Zimbabwe; 2 Department of Community Medicine, University of Zimbabwe, College of Health Sciences, P.O Box A178, Avondale, Harare, Zimbabwe; 3 Department of Physiotherapy, School of Therapeutic Sciences, Faculty of Health Sciences, University of the Witwatersrand, Johannesburg, South Africa; 4 School of Health and Rehabilitation Sciences, Faculty of Health Sciences, University of Cape Town Observatory, Cape Town, 7700 South Africa

**Keywords:** Women, Postnatal, Social support, Mental health, Zimbabwe

## Abstract

**Objective:**

Globally, 13–20% of women experience a common mental disorder (CMD) postnatally. Unfortunately, the burden of CMDs is disproportionally substantial in women from low-income countries. Nevertheless, there is a growing recognition of the buffering effect of social support (SS) on psychiatric morbidity and the need for mental well-being support services/interventions. This study evaluated the relationship between psychiatric morbidity and SS levels, and factors influencing the mental health functioning of Zimbabwean women postnatally. Data were collected from 340 mothers and were analysed through structural equation modelling.

**Results:**

The mothers’ mean age was 26.6 (SD 5.6) years. The mean Multidimensional Scale of Perceived Social Support score was 42.7 (SD 10.8), denoting high levels of SS. Additionally, 29.1% of the population reported excessive psychiatric morbidity, the median Shona Symptoms Questionnaire score was 5 (IQR: 2–8). The structural equation model demonstrated the buffering effects of SS on psychiatric morbidity (r = − 0.585, p = 0.01), and accounted for 70% of the variance. Being unmarried, increased maternal age, lower educational and income levels were associated with poorer maternal mental health. There is a need for routine; surveillance and treatment of CMDs in women in the postnatal period, including integration of low-cost, evidenced-based and task-shifting SS interventions.

**Electronic supplementary material:**

The online version of this article (10.1186/s13104-019-4151-1) contains supplementary material, which is available to authorized users.

## Introduction

Globally, between 13 and 20% of women who have just given birth experience a mental disorder [1]. Postnatal depression is particularly endemic and is a leading cause of disability in child-bearing women [2]. Other postnatal mental disorders such as anxiety, postnatal blues and psychosis are also prevalent [3]. Unfortunately, the burden of common mental disorders (CMDs) such as maternal depression is disproportionally higher in low-income countries as opposed to high-income countries with estimated prevalence rates of 19.8% and 10% respectively [4]. For example, 30–34.2% of urban-dwelling, Zimbabwean women suffer from postnatal depression (PND) [5–7]. Poverty, lower education, compromised physical health, a history of a CMD, intimate partner violence, inadequate social support, and changing cultural practices are important predictors to poor mental health status in women who have just given birth [6, 8–11].

Despite the significant burden of CMDs among women in Sub-Saharan Africa, in-depth information on mental health issues in the postnatal period is limited [7, 12]. Nevertheless, there is a growing recognition of the importance of social support (SS) in improving the mental health of women in the postnatal period [11, 13]. For instance, the buffering hypothesis postulates that effective psychological and social resources, particularly social stability, social participation, adequate emotional and instrumental support, can be considered protective, i.e. they buffer the impact of life stress on the psychological well-being of the mother [14, 15]. On the contrary, a lack of SS can lead to adverse outcomes such as; low birth weight, preterm labour, foetal neural tube defects, depression and anxiety [16]. However, there is a paucity of information on the extent to which SS influences maternal mental health in low resource-settings [7]. The current study therefore set out to identify sources of SS and evaluate the buffering effects of SS on the report of CMDs in urban-dwelling, Zimbabwean women in the postnatal period.

## Main text

### Study design, research setting and participants

We conducted a cross-sectional study at Harare City Council primary health centres. The clinics offer a variety of health services including: curative, maternity and postnatal care. Six clinics were purposively selected to ensure recruitment of participants across the socio-economic continuum. Two of the six clinics were in low to medium density catchment areas with four clinics being located in high-density suburbs [17]. Assuming a 33% prevalence of PND in urban-dwelling, Zimbabwean women [6], the minimal sample size was 340 at 95% confidence interval and 80% goal power. Women who were seeking postnatal services and willing to participate on the day of data collection were conveniently selected. Included were biological mothers ≥ 18 years with children aged 52 weeks and below. Mothers with a confirmed diagnosis of a mental health disorder and or suffering from long-term health conditions such as HIV/AIDS, cancer, among others were similarly excluded as this could have confounded the study outcomes. Mothers not proficient in either English or Shona languages were similarly excluded due to lack of financial resources for translating study outcomes into other languages.

### Study instruments

A purpose-built questionnaire was used to capture the participants’ age, gender, marital status, educational level, employment status and perceived level(s) of income. The Shona Symptom Questionnaire (SSQ), an indigenous generic screen, was used to evaluate the report of CMDs in the past 7 days. The SSQ is a binary outcome i.e. “yes” and “no” responses are scored as one and zero respectively. The score range is 0–14 and scores ≥ 8 indicate risk of CMDs. The SSQ is especially sensitive in screening for depression and anxiety and has been extensively validated in the research setting [18, 19]. The Multidimensional Scale of Perceived Social Support (MSPSS), a 12-item outcome was used to measure SS. Respondents rate the extent of satisfaction with the SS received from friends, family and significant other. Responses are ranked on a five-point Likert scale which ranges from “strongly disagree = 1” to “strongly agree = 5”. The MSPSS is one of the extensively used SS outcomes [20] and has been translated and validated into Shona (a Zimbabwean native language) [21, 22].

### Procedure

After receiving ethical and institutional approvals, the principal investigator (TK) approached prospective participants in the treatment waiting area(s). The researcher explained the study rationale, applied the selection criteria in recruiting participants and afterwards issued a detailed information sheet to mothers meeting the inclusion criteria. Mothers were obliged to provide written consent to participate in the study. All outcomes were primarily self-administered, however, the principal investigator aided participants where necessary.

### Data analysis and management

Data were entered into Microsoft Excel and analysed using STATA (Version 15). Normality was checked using the Shapiro–Wilk Test. Descriptive statistics (frequencies and means) were used to describe participants’ sociodemographics and responses on the SSQ and MSPSS. Thereafter, univariate analysis (t-tests, co-relation co-efficiencies and analysis of variance tests) was applied to determine factors influencing mothers’ mental health. Contextual factors (patients characteristics) and study primary outcomes (SSQ and MSPSS sub-scores) were then entered into the structural equation model as endogenous and exogenous variables respectively. The following parameters were set as a minimum criterion for model fit; Likelihood Ratio Chi squared Test (*χms*^2^)—criterial value: p > 0.05, root mean square error of approximation (RMSEA)—criterial value: ≤ 0.06, Comparative Fit Index (CFI)—criterial value: ≥ 0.90, Tucker–Lewis Index (TLI)—criterial value: ≥ 0.90 and the standardized root mean square residual (SRMR)—criterial value: ≤ 0.06 [23].

## Results

Many of the mothers were; married (56.8%), attained secondary education (83.4%), unemployed (65%) and reported of medium levels of income (55.3%). Their children were mostly males (50.9%), with an average age of 22.6 (SD 13) weeks. Mothers received the least and greatest amount of social support from friends and family respectively, and the mean MSPSS score was 42.7 (SD 10.8), denoting high levels of SS. Additionally, 29.1% of the mothers showed excessive psychiatric morbidity and the median SSQ score was 5 (IQR: 2–8) (Table 1). See Additional files 1 and 2 for frequencies of reported problems on the MSPSS and SSQ respectively.

**Table 1:** Participants descriptive statistics, N = 340

Variable	Attribute	Frequency, n (%)
Age of child in weeks^a^	Mean (SD)	22.6 (SD 13.0)
Gender of child	Female	167 (49.1)
	Male	173 (50.9)
Mother’s age^a^	Mean (SD)	26.6 (5.6)
Marital status	Married	193 (56.8)
	Co-habiting	101 (29.7)
	Other	46 (13.5)
Level of education	Primary	20 (5.8)
	Secondary	286 (83.4)
	Tertiary	34 (9.9)
Employment status	Formally employed	40 (11.8)
	Self-employed	77 (22.6)
	Unemployed	223 (65.0)
Perceived level of income	Below average	88 (24.1)
	Average	188 (55.3)
	Above average	70 (20.6)
Social support (MSPSS) scores^a^	Family [mean (SD)]	3.8 (SD 0.9)
	Friends [mean (SD)]	3.1 (SD 1.2)
	Significant other [mean (SD)]	3.8 (SD 1.0)
	Summative score [mean (SD)]	42.7 (SD 10.8)
Psychiatric morbidity (SSQ) scores^a^	SSQ scores ≥ 8 [n (%)]	99 (29.1%)
	Summative score: median [Q_1_–Q_3_]	5 [IQR: 2–8]

^a^Results not presented in the n (%) format

**Fig. 1:**
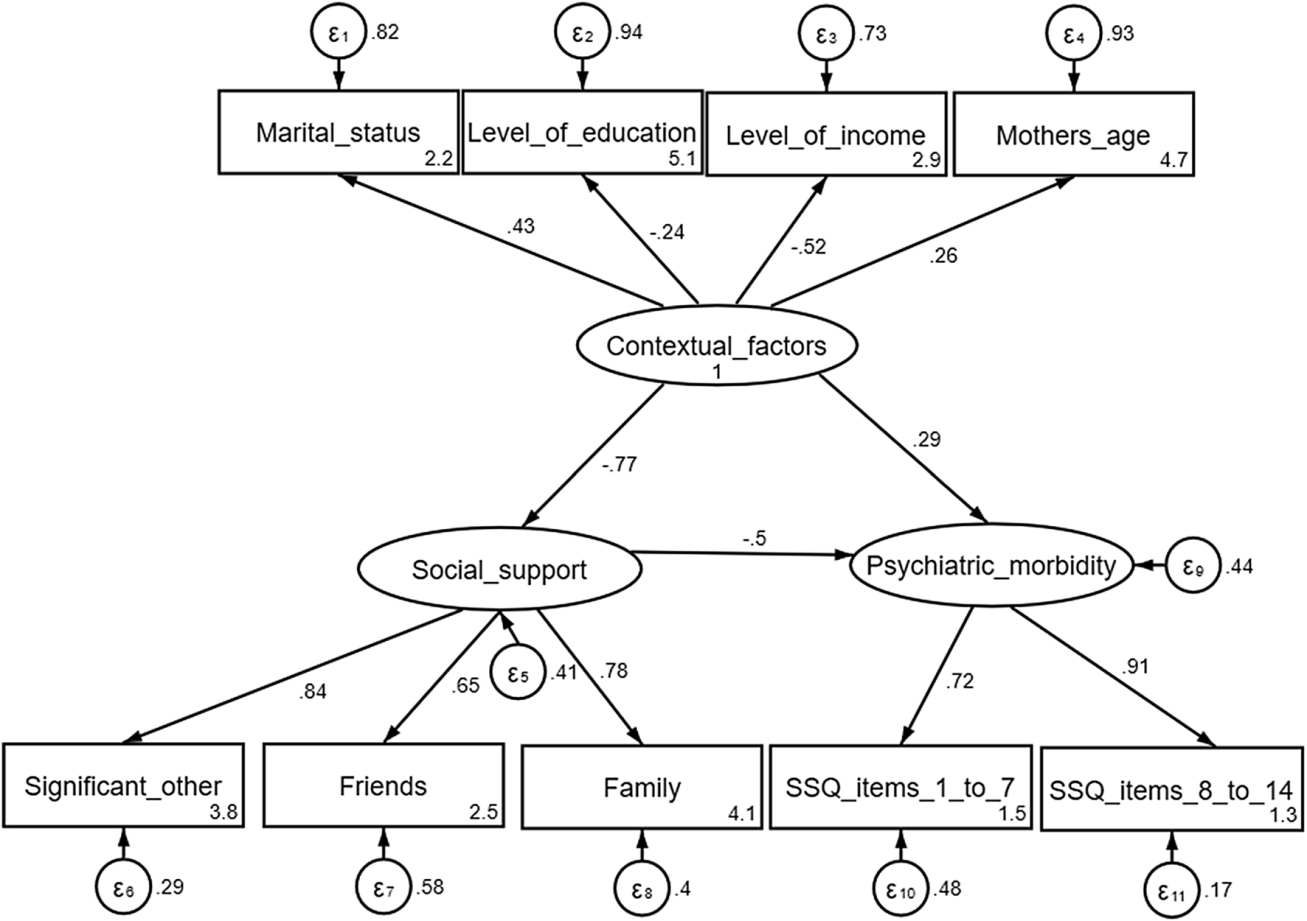
Mothers’ mental health model showing the relationship between perceived levels of social support, report of common mental disorders and contextual/demographic factors

Illustrated in Fig. 1 is the model explaining the buffering effects of social support on psychiatric morbidity (*r* = − 0.585, p = 0.01) and the associated contextual factors. The model accounted for 70% of the variance (See Additional file 3) and displayed excellent fit as outlined in Table 2. Being unmarried, lower education status, lower income level, and increased maternal age were associated with poorer maternal mental health.

**Table 2:** Model fit indices, N = 340

Fit statistic	Index	Criterion for fit	Result-interpretation
Likelihood ratio	Chi squared test (*χms*^2^)	p > 0.05	χ^2^ (df 24) = 84.87, p < 0.001—misfit
	Normed Chi square [*χ*^2^/*df*]	*χ*^2^/*df* < 2	3.5—misfit
Population error	Root mean squared error of approximation (RMSEA)-(90% CI)	RMSEA ≤ 0.06	0.054 (0.026: 0.080)—good fit
Information criteria	Akaike’s information criterion (AIC)	The smaller, the better	8965.5—best fit
	Bayesian information criterion (BIC)	The smaller, the better	9080.32—best fit
Baseline comparison	Comparative Fit Index (CFI)	CFI ≥ 0.90	0.928—good fit
	Tucker–Lewis Index (LFI)	LFI ≥ 0.90	0.893—good fit
Size of residuals	Standardized root mean squared residual (SRMR)	SRMR ≤ 0.08	0.056—good fit
	The coefficient of determination (SD)	The greater, the better	0.7—good fit

## Discussion

Consistent with previous studies, outcomes from the present study suggests that mothers who received a greater amount of SS were likely to have optimal mental health [11, 13]. Lack of SS is a demonstrated risk factor for psychiatric symptomatology in the postnatal period [24, 25]. Significant others and family were cited as the greatest sources of SS with friends providing the least support. Previous studies have shown that it is not always possible for women to differentiate the effects of spousal support from other kinship members. In collectivist cultures like Zimbabwe, the terms husband/significant other and family are habitually used interchangeably [26]. Further, mothers were likely to have decreased networking opportunities due to the demands of caring for the new infant, and this may further explain the discrepancies in sources of SS [25, 27].

The prevalence of CMDs (29.1%) was relatively higher compared to the global lifetime prevalence of 18% [28], and a 16% prevalence yielded from an almost similar, previous local study [29]. The changing patterns of mental health symptomatology in Zimbabwe especially given the advent of the HIV/AIDS pandemic and the worsening economic challenges the country has been facing may account for the dissimilarity [7]. Poverty, poor nutrition, inmate partner violence, history of depression, lack of spousal support, unstable marital status, unplanned pregnancies and increased social responsibilities are risk factors for increased psychiatric morbidity in the postnatal period for women residing in low-resource settings [11, 13, 24, 30–33].

In our study, having fewer resources (lower education and lower income), small social network (being unmarried) and maternal characteristics (increased maternal age) negatively influenced maternal mental health. Married and cohabiting mothers showed the least risk of psychiatric morbidity. Traumatic experiences such as the death of a loved one, losing a job and relationship breakdown or divorce are associated with poor mental health functioning [34–36]. These events are suggested to reflect additional stress after childbirth, at a time during which women are especially vulnerable [36, 37]. Mothers with higher levels of education reported higher levels of SS. Being educated is an important predictor to greater political and social engagement [38]. Education increases the sense of control that an individual feel over their life and concomitantly increases the chances of accessing stable relationships and expanded social networks which ultimately enhances the amount of the SS received [36, 38]. Further, educated mothers are highly likely to be employed and our findings also revealed that mothers with higher levels of perceived income indicated the least risk of psychiatric morbidity. These findings are in keeping with a previous systematic review which revealed that socio-economic disadvantaged women are five times predisposed to CMDs in the perinatal period [1].

Current evidence also suggests that increased maternal age is a risk factor for CMDs and this is in contradiction to previous studies [6, 24, 30, 32, 33]. It has been previously hypothesized that younger mothers are at an increased risk for CMDs as they may not be fully prepared for the parenting role. Further, in certain instances, the lack of SS especially spousal support, may predispose younger mothers to poor mental health functioning as some of the pregnancies maybe unplanned [24, 30]. Older mothers are likely to have greater financial resources, greater education and more likely to be mature and these are protective factors against CMDs according to the buffering hypothesis [39]. On the contrary, fertility problems, delayed parity, and prior obstetric complications are likely to predispose older mothers to CMDs [31, 40]. Further, older mothers may not receive adequate SS in comparison to first-time mothers, older mothers may be deemed “proficient” in infant care, and this predisposes them to an increased risk of CMDs [31, 40]. Other studies did not find any association between maternal age and CMDs [6, 35, 39, 41]. Considering the inconclusive evidence from literature, there is a need for further longitudinal and qualitative studies to understand the effects of maternal age on the prevalence of CMDs further.

Collectively, our study outcomes point out the need for the provision of support services such as professional counselling for the improvement of the mental health of mothers in the postnatal period. However, the lack of human resources is a massive threat towards the closure of the huge mental health treatment gap in low-resource settings [42]. This therefore calls for the integration of low-cost, evidenced-based and task-shifting interventions such as the Friendship Bench (FB) [43] in mitigating the burden of CMDs in this populace. The FB concept is centred on the use of trained, lay-persons (grandmothers) in providing standardised, problem-solving therapy (psycho-social support intervention) to persons in need of mental health services. The FB concept is in keeping with the buffering hypothesis which postulates that increased SS is associated with improved mental health [14, 15]. The FB has been successfully implemented in mitigating the effects of social stigma in individuals suffering from CMDs in the Zimbabwean context [44], and we believe the concept can be successfully integrated into routine postnatal care.

## Conclusion

The prevalence of CMDs was 29.1% and mothers who received an adequate amount of SS showed optimal mental health. Being unmarried, lower education status, lower income level, and increased maternal age were associated with poorer maternal mental health. There is need for routine surveillance and treatment of CMDs in women in the postnatal period. More importantly, there is also need for integration of low-cost, evidenced-based and task-shifting interventions such as the Friendship Bench [44] in mitigating the burden of CMDs in this populace.

## Limitations

- Causality cannot be inferred as data were collected cross-sectionally.
- Purposively selection of study sites and participants may have introduced selection bias.
- Clinical data used in applying the selection criterion were self-reported.
- Institution-based participant recruitment may have precluded selection of community-dwelling mothers at risk of poor mental health.

## Additional files

**Additional file 1.** Frequencies of responses on the MSPSS, N = 340. Table denotes frequencies of responses on the MSPSS, a 12-item social support outcome measure. Responses are rated on a five-point Likert scale, ranging from “strongly disagree = 1” to “strongly agree = 5”.

**Additional file 2.** Frequencies of responses on the SSQ, N = 340. Table denotes frequencies of responses on the SSQ, a 14-item, binary common mental disorders (CMDs) screen. Respondents indicate if they had experienced any of the enlisted symptoms in the last seven days. A yes response is scored as “one” and no as “zero”, a score ≥ 8 is indicative of risk of CMD.

**Additional file 3.** Variance explained by the model. Table denotes the variance accounted by the variables and the total model expressing the relationship between contextual factors, levels of perceived social support and report of common mental disorders.

## Abbreviations

AIDS: acquired immune deficiency syndrome
CFI: Comparative Fit Index
CMDs: common mental disorders
HIV: human immunodeficiency virus
JREC: Joint Research and Ethics Committee for the University of Zimbabwe, College of Health Sciences & Parirenyatwa Group of Hospitals
MSPSS: Multidimensional Scale of Perceived Social Support
PND: postnatal depression
RMSEA: root mean square error of approximation
SD: standard deviation
SRMR: standardized root mean square residual
SS: social support
SSQ: Shona Symptom Questionnaire
TLI: Tucker–Lewis Index
