# Supplementary material for: A structural equation modelling of the buffering effect of social support on the report of common mental disorders in Zimbabwean women in the postnatal period
Source: BMC Res Notes. 2019 Feb 28;12:110. doi: 10.1186/s13104-019-4151-1 (PMC6394011; doi:10.1186/s13104-019-4151-1)
Supplement: Supplementary file 1 — Additional file 1. Frequencies of responses on the MSPSS, N = 340. Table denotes frequencies of responses on the MSPSS, a 12-item social support outcome measure. Responses are rated on a five-point Likert scale, ranging from “strongly disagree = 1” to “strongly agree = 5”. [file 13104_2019_4151_MOESM1_ESM.docx]

**Additional file 1: Frequencies of responses on the MSPSS, N=340**

| Item | Strongly Disagree, n (%) | Disagree, n (%) | Neutral, n (%) | Agree, n (%) | Strongly Agree, n (%) |
| --- | --- | --- | --- | --- | --- |
| 1. Special person in need | 35 (10.3) | 39(11.5) | 59(17.4) | 114(33.5) | 93(27.4) |
| 1. Special person joys and sorrows | 20 (5.9) | 32(9.4) | 44(12.9) | 133(39.1) | 111(32.6) |
| 1. Family help | 26 (7.6) | 24(7.1) | 40(11.8) | 136(40.0) | 114(33.5) |
| 1. Family support | 24 (7.1) | 27(7.9) | 55(16.2) | 126(37.1) | 108(31.8) |
| 1. Special person comfort | 24 (7.1) | 25(7.4) | 43(12.6) | 135(39.7) | 113(33.2) |
| 1. Friends help | 69 (20.3) | 42(12.4) | 69(20.3) | 97(28.5) | 63(18.5) |
| 1. Counting on friends | 98(28.8) | 54(15.9) | 76(22.4) | 73(21.5) | 39(11.5) |
| 1. Family problems | 11(3.2) | 33(9.7) | 38(11.2) | 145(42.6) | 113(33.2) |
| 1. Friends joys and sorrows | 51(15.0) | 43(12.6) | 69(20.3) | 98(28.8) | 79(23.2) |
| 1. Special person feelings. | 22(6.5) | 23(6.8) | 40(11.8) | 133(39.1) | 122(35.9) |
| 1. Family decisions | 27(7.9) | 26(7.6) | 47(13.8) | 134(39.4) | 106(31.2) |
| 1. Friends problems | 75(22.1) | 41(12.1) | 68(20.0) | 94(27.6) | 62(18.2) |
