# Supplementary material for: A structural equation modelling of the buffering effect of social support on the report of common mental disorders in Zimbabwean women in the postnatal period
Source: BMC Res Notes. 2019 Feb 28;12:110. doi: 10.1186/s13104-019-4151-1 (PMC6394011; doi:10.1186/s13104-019-4151-1)
Supplement: Supplementary file 2 — Additional file 2. Frequencies of responses on the SSQ, N = 340. Table denotes frequencies of responses on the SSQ, a 14-item, binary common mental disorders (CMDs) screen. Respondents indicate if they had experienced any of the enlisted symptoms in the last seven days. A yes response is scored as “one” and no as “zero”, a score ≥ 8 is indicative of risk of CMD. [file 13104_2019_4151_MOESM2_ESM.docx]

**Additional file 2: Frequencies of responses on the SSQ, N=340**

|  | Response | |
| --- | --- | --- |
| Item | **Yes, n (%)** | **No, n (%)** |
| 1. Thinking pattern | 199(58.5) | 141(41.5) |
| 1. Concentration | 167(49.1) | 173(50.9) |
| 1. Temper | 162(47.6) | 178(52.4) |
| 1. Dreams | 136(40.0) | 204(60.0) |
| 1. Hallucinations | 25(7.4) | 315(92.6) |
| 1. Stomach ache | 130(38.2) | 210(61.8) |
| 1. Fright | 59(17.4) | 281(82.6) |
| 1. Insomnia | 146(42.9) | 194(57.1) |
| 1. Crying | 183(53.8) | 157(46.2) |
| 1. Tired | 176(51.8) | 164(48.2) |
| 1. Suicidal ideation | 29(8.5) | 311(91.5) |
| 1. Unhappy | 117(34.4) | 223(65.6) |
| 1. Work lagging | 107(31.5) | 233(68.5) |
| 1. Decision making | 141(41.5) | 199(58.5) |
