# Supplementary material for: A structural equation modelling of the buffering effect of social support on the report of common mental disorders in Zimbabwean women in the postnatal period
Source: BMC Res Notes. 2019 Feb 28;12:110. doi: 10.1186/s13104-019-4151-1 (PMC6394011; doi:10.1186/s13104-019-4151-1)
Supplement: Supplementary file 3 — Additional file 3. Variance explained by the model. Table denotes the variance accounted by the variables and the total model expressing the relationship between contextual factors, levels of perceived social support and report of common mental disorders. [file 13104_2019_4151_MOESM3_ESM.docx]

**Additional file 3: Variance explained by the model**

|  |  |  | Variance |  |  |  |  |
| --- | --- | --- | --- | --- | --- | --- | --- |
|  |  | **fitted** | **predicted** | **residual** | **R^2^** | **mc** | **mc^2^** |
| Observed variable | MSPSS Significant other subscale | 1.007 | 0.719 | 0.288 | 0.714 | 0.845 | 0.714 |
|  | MSPSS Family subscale | 0.896 | 0.539 | 0.357 | 0.602 | 0.776 | 0.602 |
|  | MSPSS Friends subscale | 1.524 | 0.648 | 0.876 | 0.425 | 0.652 | 0.425 |
|  | SSQ items 1-7 | 3.067 | 1.580 | 1.487 | 0.515 | 0.718 | 0.515 |
|  | SSQ items 8-14 | 4.229 | 3.517 | 0.713 | 0.832 | 0.912 | 0.832 |
|  | Marital status | 0.516 | 0.094 | 0.422 | 0.182 | 0.426 | 0.182 |
|  | Level of income | 0.446 | 0.120 | 0.326 | 0.268 | 0.518 | 0.268 |
|  | Level of education | 0.157 | 0.009 | 0.148 | 0.058 | 0.242 | 0.058 |
|  | Mothers’ age | 31.466 | 2.053 | 29.413 | 0.065 | 0.255 | 0.065 |
| Latent variables | MSPSS summative scores | 0.719 | 0.421 | 0.297 | 0.586 | 0.766 | 0.586 |
|  | SSQ summative scores | 1.580 | 0.883 | 0.698 | 0.559 | 0.747 | 0.559 |
|  | **Overall variance explained by the model** | | | | **0.700** |  |  |
